# Modeling abundance and risk impact of *Vespa velutina nigrithorax* (Hymenoptera: Vespidae) in Korea: application of a species abundance model

**DOI:** 10.1038/s41598-023-40016-9

**Published:** 2023-08-21

**Authors:** Min-Jung Kim, Seongbin Bak, Chuleui Jung

**Affiliations:** 1 Forest Entomology and Pathology Division, National Institute of Forest Science, Seoul, 02455 Republic of Korea; 2 Department of Plant Medicals, Andong National University, Andong, 36729 Republic of Korea; 3 Agricultural Science and Technology Research Institute, Andong National University, Andong, 36729 Republic of Korea

**Keywords:** Ecology, Ecological modelling

## Abstract

The Asian yellow-legged hornet, *Vespa velutina nigrithorax*, is native to Southeast Asia. However, it has invaded many countries in temperate regions, causing serious threats to honeybees and human health. With a growing demand for estimating the potential distribution of this species, multiple studies have resorted to occurrence-based models. However, they are less informative for predicting local abundance patterns associated with the species’ impact. Thus, we aimed to develop an abundance-based distribution model for *V. v. nigrithorax* in Korea to support the forecast of its impact and associated management strategies. The abundance data of *V. v. nigrithorax* were collected from 254 sites for 4 years covering the country and used to develop a model with bioclimatic and land composition variables. Along with the abundance model, the classical occurrence model was tested to determine whether it could provide a reasonable prediction on the estimation of local abundance. As a result, the abundance model provided higher discriminative power and accuracy than the occurrence model to evaluate the impacts caused by *V. v. nigrithorax*. On the other hand, the occurrence model was not able to discriminate abundance in the areas occupied by *V. v. nigrithorax*, indicating an unclear occurrence-abundance relationship or oversimplification of the estimated niche created by the occurrence model. Based on the final abundance model, risk indices for human health and honeybee losses were suggested. These results could help to provide support for risk management of *V. v. nigrithorax* in Korea and to give biological information to other countries where this species has already become established or which it is likely to invade in the near future.

## Introduction

The Asian yellow-legged hornet, *Vespa velutina nigrithorax* (Hymenoptera: Vaspidae), is native to Southeast Asia^1^. However, it has since invaded many countries in temperate regions, including those of East Asia and Central and Western Europe^2–5^. *Vespa velutina nigrithorax* is a generalist predator that preys on various groups of invertebrates such as honeybees, wild bees, flies, and social wasps to maintain its colony^6,7^. Its predatory nature, combined with a high reproductive ability as a social wasp, could pose a significant threat to other insect groups in the regions it has invaded^5,7–9^. Rome et al.^7^ estimated that a single colony of *V. v. nigrithorax* requires an average of 11.32 kg of insect biomass to complete its life cycle in France, which is roughly equivalent to over 90,000 honeybee-like prey items. Although its impact varies depending on the surrounding habitat and food availability, this species is generally considered as problematic in the beekeeping industry due to its frequent predation of honeybees^7,10–13^. The estimated annual cost of managing hornet species in Korean apiaries exceeds $60,000,000, with *V. v. nigrithorax* contributing significantly to this expense^12^.

In addition to its impact on beekeeping, *V. v. nigrithorax* has been speculated to negatively affect native biodiversity and ecosystems. Numerous researchers have suggested that the decline of a competing congeneric species, *Vespa simillima*, is due to the establishment of *V. v. nigrithorax* in Korea^8,14–17^. Due to the potential impacts of *V. v. nigrithorax* on biodiversity in invaded regions, this species has been included on the EU's list of Invasive Alien Species (IAS) of Union concern (the Union list) under Regulation EU 1141/2016. The Korean Ministry of Environment (KME) also designated this species as an ecological disturbance species in 2019 (http://me.go.kr/). The potential sting risk to citizens and the consequent removal efforts of hives represent additional concerns in public health, as *V. v. nigrithorax* is frequently found in the vicinity of residential areas, unlike other hornet species^14,18,19^. Accidents, and even fatal incidents, have been reported from countries where *V. v. nigrithorax* has successfully established^20,21^. The Korean National Fire Agency (NFA) reported that over 16,000 of hive removal requests were specifically for *V. v. nigrithorax* in 2020 (https://www.nfa.go.kr/).

As *V. v. nigrithorax* has rapidly expanded its range in non-native countries, it is crucial to identify suitable habitats for this species in order to evaluate the risk of its establishment. In line with this need, multiple studies have suggested its potential distribution adopting species distribution models (SDMs) based on its occurrence records (presence and background or pseudo-absence)^22–28^. These studies have consistently shown that *V. v. nigrithorax* can establish itself in most of temperate regions across Northern and Southern hemispheres, including East Asia, Europe, and some parts of North and South America, Australia, Africa, and the Pacific. This information could be used to assess its establishment risk in non-invaded regions. However, the results of SDMs provide limited guidance for planning risk management against the hornet’s impact in countries like Korea, where *V. v. nigrithorax* has successfully established itself across the entire territory. This limitation arises because occurrence-based models (i.e., classical SDMs) only yield the probability of establishment or standardized habitat suitability, not the abundance of the species within individual grid cells, which is crucial for understanding potential impacts^29–31^. Moreover, no definitive evidence suggests a linear correlation between local species abundance and the outcomes of SDMs^31–35^. As a result, directly predicting abundance (i.e., species abundance models, SAMs) provides a more intuitive assessment of the impact of well-established invaders within target regions^31,36^.

However, accurately predicting a species’ abundance has to overcome several hurdles. Abundance data are generally scarce in public databases of species occurrence records^33^. Thus, estimation of the target species density across the study area often becomes a prerequisite for developing SAMs. This step would be a key for a realistic model output, because extrapolations of SAMs frequently result in poor predictions with high uncertainties^34^. In addition, for certain species that occupy the whole study area, as is the case of *V. v. nigrithorax* in Korea, accurate predictions of a species’ abundance could be challenging within the given environmental space^37^. Species abundance cannot be well described by climatic variables alone, which are commonly used in SDMs studies. The estimated climatic niche could overlook other confounding factors such as interspecific interaction and resource availability in relation to local demographic processes to determine the abundance of a species^33,38^. These obstacles are more prominent in SAMs than in the SDMs studies^34^. Therefore, it is necessary to obtain reliable abundance data across the study area and input environmental variables associated with biological characteristics of the target species in order to predict its abundance more accurately based on environmental niche theory^39^.

The aim of this study was to develop SAMs for *V. v. nigrithorax* in Korea to support the forecast of its impact and management strategies. To achieve this, we first constructed abundance data of *V. v. nigrithorax* across the whole territory of Korea and then tried to develop SAMs using two machine learning algorithms with environmental variables including bioclimate and landscape composition. Additionally, we empirically tested whether classical SDMs based on occurrence records could generate proper predictions in estimating abundance. Finally, we proposed risk indices related to public health and honeybee loss at the city or county level of Korea by synthesizing these results.

## Material and methods

### Abundance data

**Figure 1:**
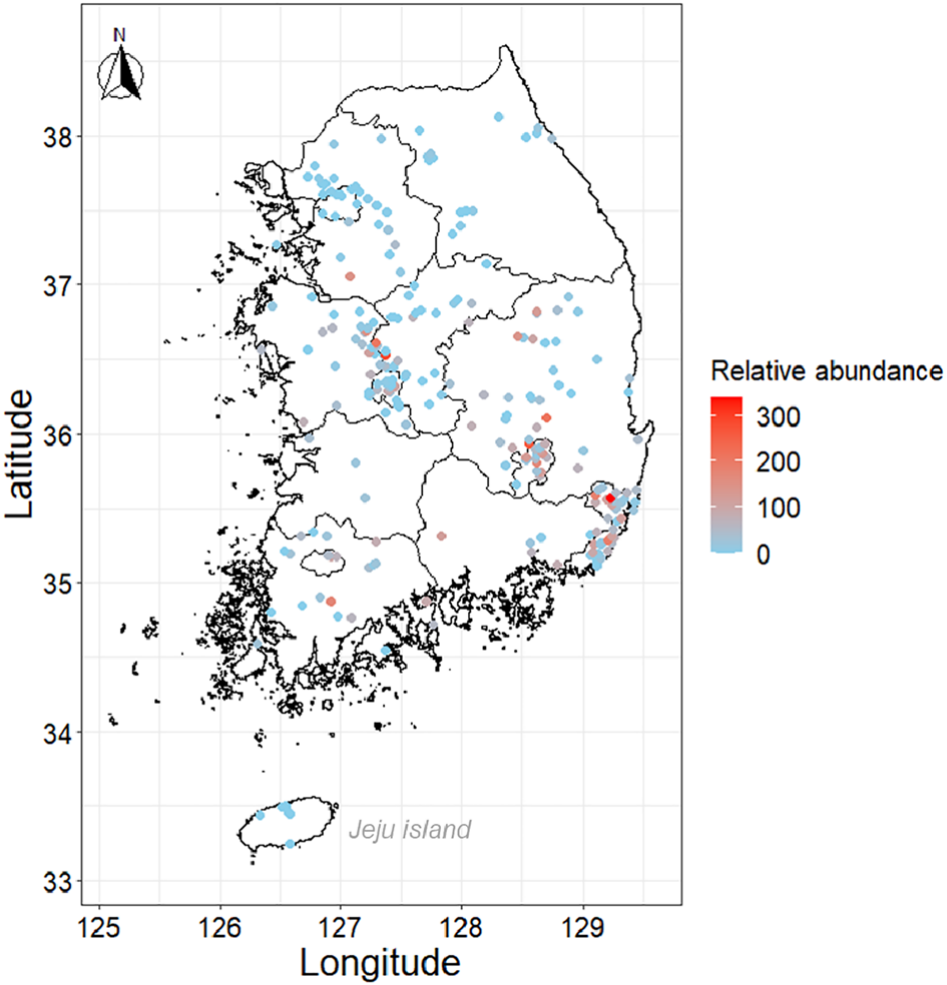
Sampled location for estimating the relative abundance of *Vespa velutina nigrithorax* in Korea during 2018–2021.

Local abundance of *V. v. nigrithorax* was investigated in 254 locations nationwide from 2018 in cooperation with members of Korea’s beekeeping association (KBA) (https://www.korapis.or.kr/) (Fig. 1). Given that *V. v. nigrithorax* had already spread to the furthest administrative areas from the regions where it was first reported in 2003 at the beginning of this study^2,25^, we assumed that this hornet species had reached an equilibrium state, a precondition for the estimation of ecological niche^30^. Sampling was conducted for 1 month (September or October), during the active foraging season of *V. v. nigrithorax*^7,11,13,20^. A wing-shaped trap baited with fermented oak sap (DamokEcotech; Sangju; Korea) was used at each site. Although this trap is not specifically designed for *V. v. nigrithorax*, a high capture efficiency for our target species was reported in a previous study^40^. The collected samples were identified at the species level according to the morphological key of Vespidae^3,14,41–44^. The field sampling was performed for at least 1 year (over multiple years for some locations with a maximum of 4 years) during 2018–2021. Specifically, 239 locations were sampled in 2018, 108 locations in 2019, 80 locations in 2020, and 56 locations in 2021 (Supplementary Fig. 1A).

We assumed that the number of captured individuals represents the foraging abundance, which is a result of a complex interaction of various components such as preferred nesting location, population size, and food availability. Thus, we considered the trap catches as an indicator of the relative abundances of *V. v. nigrithorax* in sampling locations. For site sampled over multiple years, the trap catches of each year were averaged. During our investigation, *V. v. nigrithorax* was not captured in Jeju Island, the southernmost part of Korea. Records in Jeju (six points) were excluded from the abundance database, as it was unclear whether the absence of this species was due to geographic or ecological barriers^12,45^. A total of 248 records were compiled to represent abundance data of *V. v. nigrithorax* (Supplementary Fig. 1B).

### Covariates

Of a subset of 19 bioclimatic variables, 13 with 1 km^2^ spatial resolution were obtained from the WorldClim website (https://www.worldclim.org/), referring to previous SDMs studies for *V. v. nigrithorax* (Table 1). Landcover compositions were also considered as environmental variables for developing SAMs^24,28^. We expected that landcover composition could affect the location of nest building of *V. v. nigrithorax* and consequent occurrence of foraging worker^7,38^. Sauvard et al.^46^ have evaluated flight ability of *V. v. nigrithorax* workers and found that workers mainly forage within a 1 km radius around their hive. We thus created surrounding land type variables to include the area of each type within a 1 km radius around a specific location. In other words, areas of surrounding land types (< 1 km) were attributed to a gridded cell. A landcover layer with 30 m^2^ spatial resolution including seven land classes was obtained from KME (https://egis.me.go.kr/) and used to create land type variables. All gridded variable layers were matched to bioclimatic variables across the study area using a resampling technique provided by ArcGIS Pro^47^. A total of 20 environmental variables including 13 bioclimatic and seven land types were prepared to develop SAMs for *V. v. nigrithorax*.

**Table 1:** Summary of species distribution modeling studies adopting a correlative approach for predicting potential distribution of *Vespa velutina nigrithorax.*

Modeling algorithm		Occurrence data	Spatial extent	Variable		References
Statistical regression	Machine learning			Bioclimatic^a^	Others	
GLM, GAM, MARS, FDA	CTA, ANN, GBM, RF	Presence/pseudo-absence	Global	Bio1, Bio4, Bio5, Bio6, Bio12, Bio13, Bio14, Bio15	–	^22^
GLM	–	Presence/pseudo-absence	Iberian Peninsula	Bio1, Bio3, Bio7, Bio13	Urban settlement, Agricultural field, Scrubland, NDVI, Distance to river	^24^
GLM, GAM, MARS, FDA	CTA, ANN, GBM, RF	Presence/pseudo-absence	Global	Bio1, Bio4, Bio5, Bio6, Bio12, Bio13, Bio14, Bio15	–	^26^
–	MaxEnt	Presence/background	Global	Bio1, Bio11, Bio12, Bio14	–	^27^
GLM, GAM, MARS, FDA	CTA, ANN, GBM, RF, MaxEnt	Presence/background	Mediterranean islands	Bio3, Bio15, Bio18, Bio19	Continentality, Slope, Landcover, Human footprint	^28^

*GLM* generalized linear model, *GAM* generalized additive model, *MARS* multivariate adaptive regression spline, *FDA* flexible discriminant analysis, *CTA* classification tree analysis, *ANN* artificial neural network, *GBM* gradient boosting machine, *RF* random forest.

^a^Description of bioclimatic variables are given in the WorldClim website (https://www.worldclim.org/).

To avoid multi-collinearity, we initially examined all-pairwise correlations of 20 candidate variables and filtered out variables correlated with each other (Pearson’s r > 0.8). Among correlated variants in each pair, one variable was selected based on its frequency of use in previous studies (Table 1). Consequently, we incorporated the following 13 variables to construct a covariate space: annual mean temperature (Bio1), isothermality (Bio3), annual precipitation (Bio12), precipitation of wettest month (Bio13), precipitation of driest month (Bio14), precipitation seasonality (Bio15), agricultural land (Prop_agri), barren land (Prop_barren), forest (Prop_forest), grassland (Prop_grass), urban area (Prop_urban), water area (Prop_water), and wetland (Prop_wet) (Table 2).

**Table 2:** Variables used in environmental niche modeling for* Vespa velutina nigrithorax.*

Factor	Variable	Description	Mean (max–min)	Unit
Bioclimate	Bio1	Annual mean temperature	11.74 (17.41–2.75)	°C
	Bio3	Isothermality	28.31 (33.41–17.60)	–
	Bio12	Annual precipitation	1363 (1928–1073)	mm
	Bio13	Precipitation of wettest month	345 (486–209)	mm
	Bio14	Precipitation of direst month	24 (77–14)	mm
	Bio15	Precipitation seasonality	94.89 (116.07–35.66)	–
Surrounding land type (< 1 km radius)	Prop_agri	Agricultural land area	0.59 (3.18–0.00)	km^2^
	Prop_barren	Barren land area	0.04 (2.55–0.00)	km^2^
	Prop_forest	Forest area	2.16 (3.25–0.00)	km^2^
	Prop_grass	Grassland area	0.09 (2.63–0.00)	km^2^
	Prop_urban	Urban area	0.17 (3.20–0.00)	km^2^
	Prop_water	Water area	0.05 (2.97–0.00)	km^2^
	Prop_wet	Wetland area	0.02 (1.78–0.00)	km^2^

### Modeling procedure

We adopted machine-learning approaches to develop SAMs, accommodating complex nonlinear responses and interaction in relation of abundance^29,48^. Among a variety of modeling algorithms, two different non-parametric models, Random Forest (RF) and Gradient Boosting Machine (GBM)^49,50^, were used. RF and GBM are tree-based models using bagging and boosting algorithms to predict response variables, respectively. These models have demonstrated good performance in predicting species abundance in previous study^34^.

Before running the model, we categorized the abundance data into four groups based on the average number of individuals captured at the sampling locations (Absence: 0, Low: 1–9, Mid: 10–99, and High: ≥ 100). These data were then randomly split into training (70%) and test (30%) sets while maintaining the same compositional proportion of categories in each set (i.e., stratified data partitioning to escape biased division). Training data, referenced with 13 environmental variables, were fitted using the RF and GBM methods, implemented in *randomForest* and *gbm* packages in R 4.1.1, respectively^51^. The models were run with default settings provided by the R packages, except for the number of trees in RF model (ntree = 1000, in *randomForest* function), which determines the number of base classifiers used to create an ensemble prediction. As a response variable of SAMs, original mean abundance estimates ($x$) and their log transformation ($\mathrm{log}(x+1$)) were used. Thus, a total of four model variants were examined in this study to develop SAMs: two modeling algorithms (RF and GBM), each paired with two types of response variable. Modeling procedures, including data partitioning, were replicated 100 times independently.

Each model was evaluated using test data based on evaluation metrics, accuracy and discrimination power, suggested by Norberg et al.^52^ and Waldock et al.^34^. Accuracy indicates the degree of closeness between the model's predictions and observations in the test set. We measured accuracy as the mean absolute error between predicted and observed values from the test data. This accuracy value was then scaled by dividing mean observed abundance to allow for a direct comparison of the results. Discrimination refers to the degree of consistency between prediction and observation, regardless of its magnitude. We used two statistics, Pearson’s correlation coefficient (Pearson’s r) and the slope of a linear model between predicted and observed abundance, to evaluate discriminative power. Thus, target values for accuracy and discrimination in an ideal model would be 0 and 1, respectively. Model evaluation statistics were aggregated and averaged to select the best options for estimating the abundance of *V. v. nigrithorax*. The best model was selected based on the sum of ranks of each evaluation statistic.

In addition to the SAMs, we empirically tested the performance of classical SDMs in predicting the abundance. Binary-encoded occurrence data (presence: 1, absence: 0) transformed from abundance data were used to fit the model with RF and GBM algorithms. A total of 100 replicated runs were performed with the same data-partitioning method with SAMs training. Accuracy and discrimination were then assessed. Because SDMs generate the probability of establishment ranging from 0 to 1, we compared the result with the relative abundance scaled by the maximum abundance values. This provided valuation statistics representing the model’s applicability in estimating *V. v. nigrithorax* abundance. Additionally, we calculated the AUC (area under the receiver operating characteristic) value, representing classification ability from the binary response. This metric is one of the important evaluation tools for determining performance in most of SDMs studies. Among RF and GBM models adopting an SDM approach, the model with a higher mean AUC score was chosen^30^.

Final models of SAMs and SDMs were constructed based on selected model variants using whole abundance and occurrence responses, respectively, without data partitioning. To compare the discriminative performance of the two models, predictions generated from both were classified according to the four observed abundance categories (Absence: 0, Low: 1–9, Mid: 10–99, and High: ≥ 100). Variations in these classified predictions relative to the true abundance levels were assessed using Kruskal–Wallis rank sum test, followed by a post-hoc comparison with Bonferroni correction using *dunn.test* packages in R 4.1.1^51^. The marginal effect of each variable in the final SAMs was depicted by a partial dependence plot to identify density response according to the environmental gradient. The projected final SAMs, transformed to a log scale ($\mathrm{log}(x+1$)), was aggregated as the city (-si) or county (-gu) level of the administrative division of Korea by averaging predicted values in ArcGIS pro^47^.

### Risk index

We obtained geocoded records of *V. v. nigrithorax* nest removal in 2020 from the National Fire Agency, Korea (NFA; https://www.nfa.go.kr/) (Supplementary Table 1). Since NFA data were accumulated by removal actions following citizens’ call reports, we considered these records would be influenced by the detectability of a *V. v. nigrithorax* and its abundance as well. Thus, we first aggregated NFA data into the same administrative unit of a predicted mean abundance map for *V. v. nigrithorax* and standardized the number of records by dividing the residential population of each geographic unit (https://kosis.kr/) (Supplementary Table 1). This procedure produced a map of the number of call reports for nest removal per residential population attributed to each administrative unit. We expected this standardized NFA data to have a closer relationship with the abundance of *V. v. nigrithorax* in a given area. In addition to the NFA data, we collected information about the appearance rate of *V. v. nigrithorax* in apiaries in the province (-do) level of Korea^12^. This data was constructed through questionnaires answered by beekeepers to investigate the appearance of hornet species in Korea, including % rate of each species in apiaries in a given administrative unit. More than half of the responding beekeepers (n = 225) answered that *V. v. nigrithorax* occurred in their apiaries.

We checked whether our abundance prediction for *V. v. nigrithorax* could explain risks for public health and honeybee losses. For this, we conducted a correlation analysis between predicted abundance, and standardized NFA and questionnaire data. We assumed that the magnitude of risk was the function of hazard (*V. v. nigrithorax* abundance) and sensitivity (residential population or number of honeybee colonies) (Supplementary Table 1). Therefore, risk indices for public health and honeybee losses were calculated by multiplying predicted abundance by each sensitivity factor. Risk indices were projected as the city or county level of Korea in ArcGIS pro^47^.

## Results

The estimated abundance of *V. v. nigrithorax* across Korea showed a positively skewed distribution (Supplementary Fig. 1B). The abundance of the hornet varied by sampling location, with no captures at 61 locations, low abundance (1–9 captures) at 50 locations, mid-range abundance (10–99 captures) at 110 locations, and high abundance (≥ 100 captures) at 27 locations. The maximum average capture was 338.5. Performances of SAMs varied depending on both modeling algorithms and response transformation (Table 3). Among the models studied, the RF model, using a log-transformed response, consistently showed the best performance in all evaluation statistics based on results of 100 replicated runs. Thus, this model variant was finally selected to predict the abundance of *V. v. nigrithorax*. In SDMs, the RF model showed better and more reasonable predictive power (AUC > 0.7) for distinguishing presence and pseudo-absence points than the GBM, based on the mean AUC score. However, SDMs poorly performed, particularly regarding accuracy, for predicting abundance (Table 3).

**Table 3:** Mean evaluation statistics (standard deviation) of species abundance models (SAMs) and species distribution models (SDMs) for *Vespa velutina nigrithorax* for selecting the best model variant.

Response variables	Model	Transformation	Accuracy^a^	Discrimination		AUC	Final selection
				Correlation coefficient^b^	Slope^c^		
Abundance (SAMs)	RF	None	0.956 (0.0802)	0.271 (0.0933)	0.557 (0.2270)	–	
		Log	0.537 (0.0388)	0.470 (0.0760)	0.867 (0.1809)	–	Selected
	GBM	None	1.114 (0.1042)	0.158 (0.0986)	0.234 (0.1501)	–	
		Log	0.599 (0.9600)	0.344 (0.2700)	0.477 (0.5600)	–	
Occurrence (SDMs)	RF	Presence (1)/absence (0)	5.973 (0.4952)	0.299 (0.0839)	0.235 (0.0776)	0.732 (0.0537)	Selected
	GBM		6.145 (0.5713)	0.175 (0.1132)	0.094 (0.0609)	0.649 (0.0617)	

^a^Standardized mean absolute error.

^b^Pearson’s r coefficient correlation between prediction and observation.

^c^Slope of linear model between prediction and observation.

**Figure 2:**
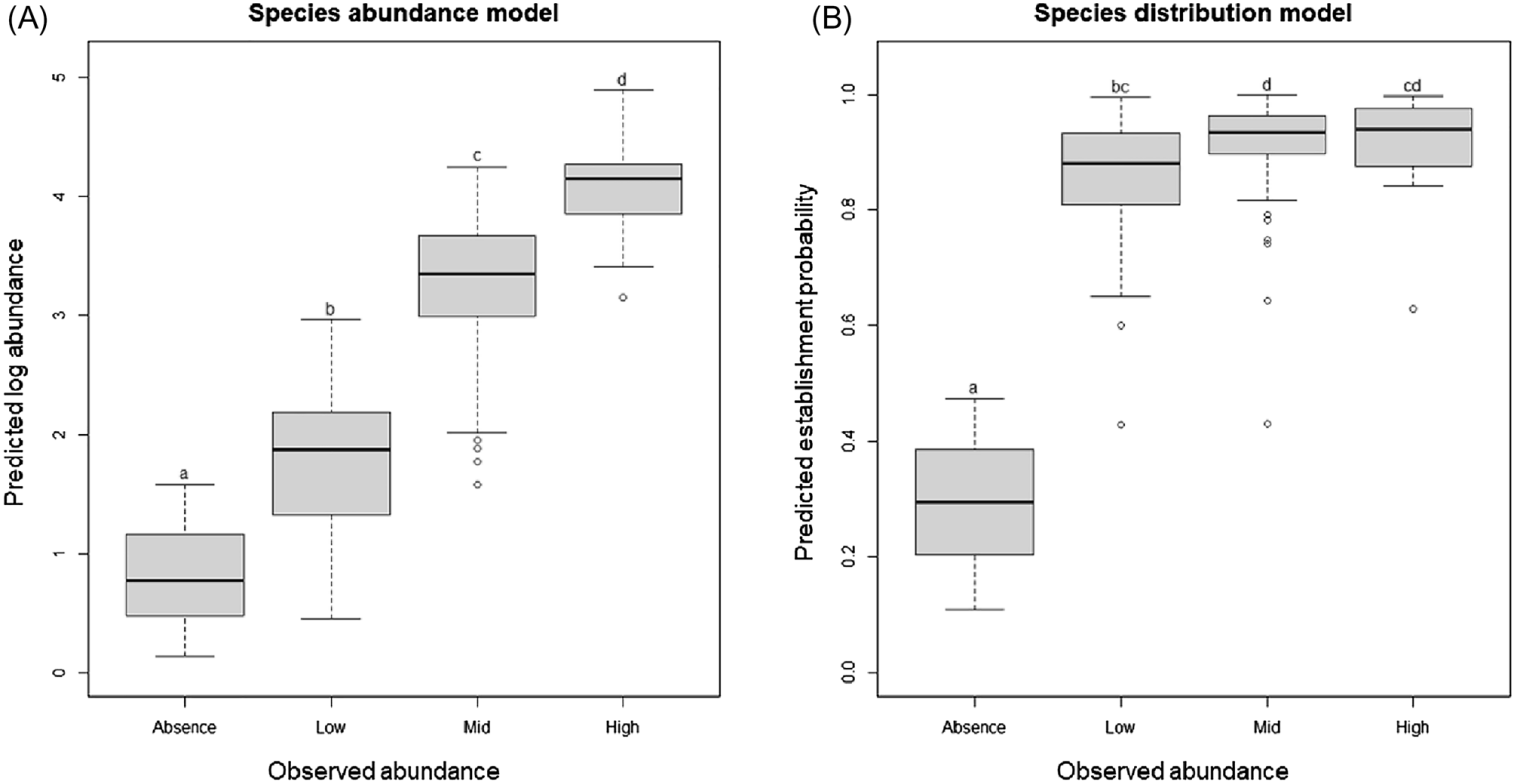
Distribution of predicted values from (**A**) the species abundance models (SAMs) and (**B**) the species occurrence models (SDMs) according to observed abundance levels of *Vespa velutina nigrithorax* in Korea. Observed abundance was categorized as Absence (0), Low (1–9), Mid (10–99), and high (≥ 100) based on actual number of captured individuals. Different letters above the bars represent significant differences in predicted values among the categories, as determined by the Kruskal–Wallis rank sum test with Bonferroni correction (adjusted *p* < 0.05).

**Figure 3:**
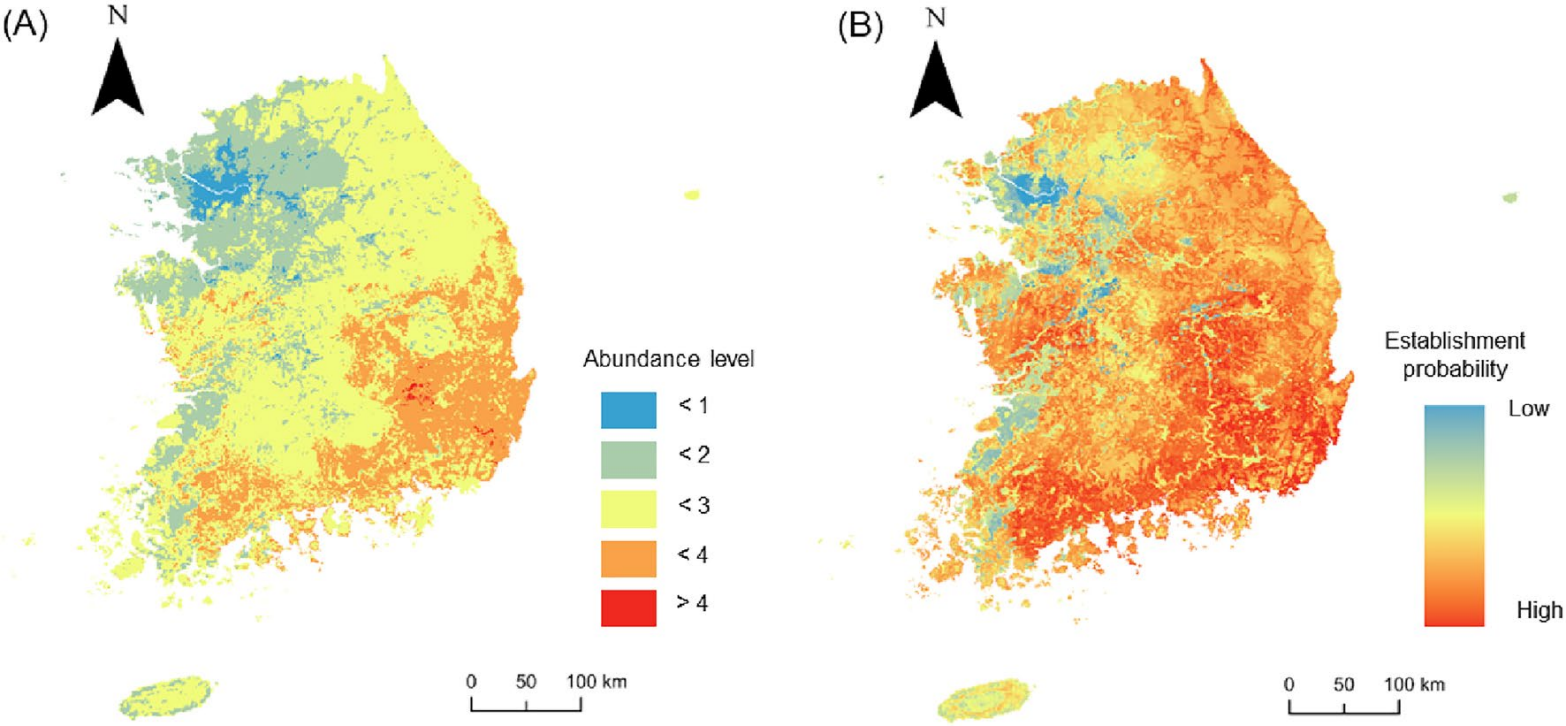
Predicted distribution map of (**A**) abundance and (**B**) establishment probability of *Vespa velutina nigrithorax* in Korea.

The final models of SAMs and SDMs demonstrated different performance in predicting the abundance of *V. v. nigrithorax*. Predictions based on SAMs could discriminate regional abundance levels of *V. v. nigrithorax* (χ^2^ = 193.69, df = 3, *p* < 0.0001) (Fig. 2A) (Supplementary Fig. 2A). The predicted establishment probability from the SDMs could also distinguish absence points from presences (χ^2^ = 143.39, df = 3, *p* < 0.0001). However, it failed to differentiate density levels of *V. v. nigrithorax* within the occupancy area (Fig. 2B) (Supplementary Fig. 2B). Both models predicted values of higher abundance or establishment probability of *V. v. nigrithorax* in southeastern parts of Korea, while scores were lower in the Northwest (Fig. 3). However, the SDM generated relatively higher predictive values than the SAM across the whole study area, but it could not discriminate regional abundance of *V. v. nigrithorax* (Figs. 2, 3).

**Figure 4:**
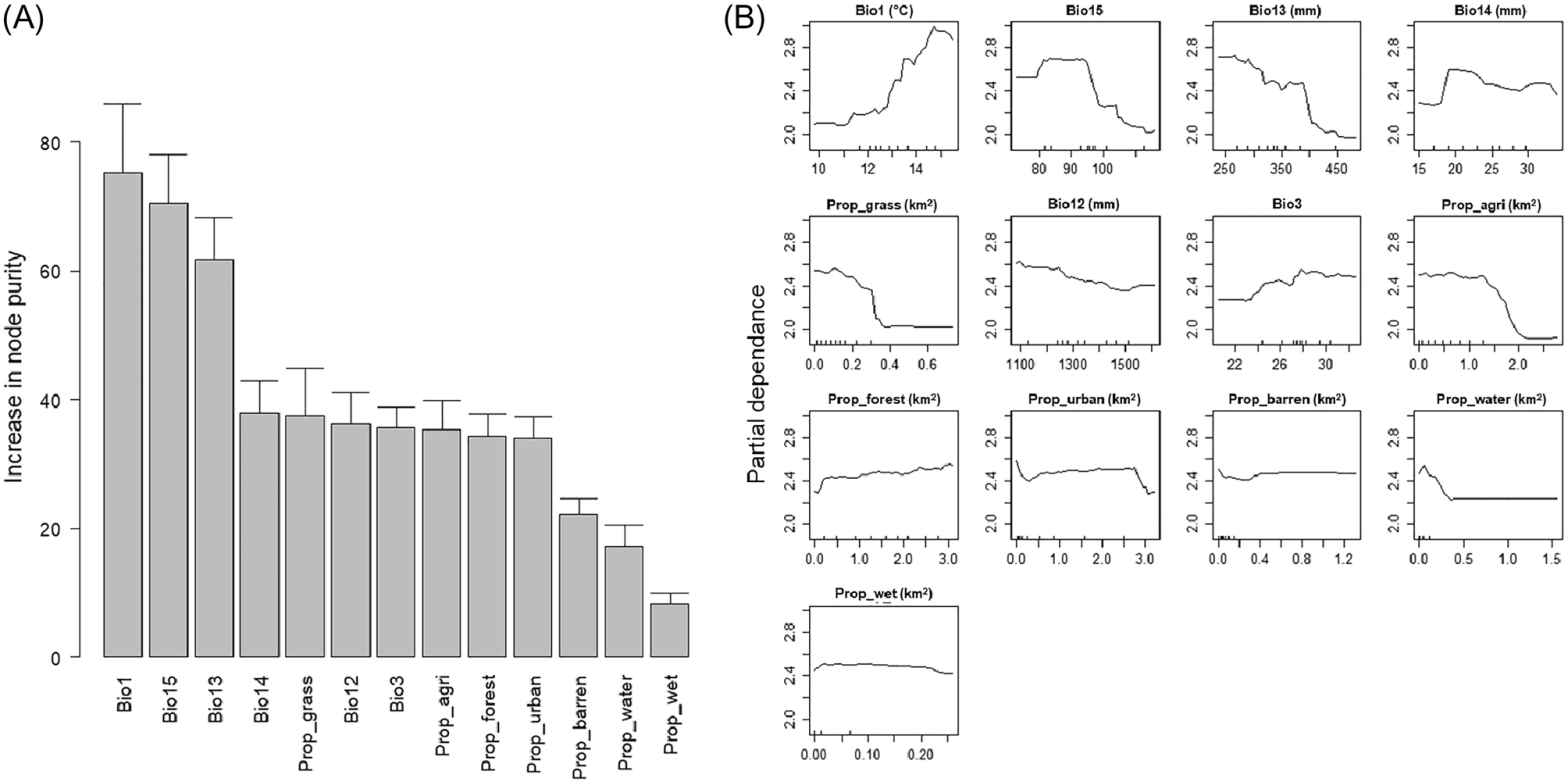
(**A**) Relative importance and (**B**) partial dependences of environmental variables in the SAMs for *Vespa velutina nigrithorax* in Korea. Importance was evaluated as the magnitude of increase in node purity (mean ± S.D.). Partial dependence represents a marginal effect of a variable when others are held constant, indicating the change of abundance prediction relative to the environmental gradient. Descriptions and ranges of each variable could be found in Table 2.

Based on the contribution of each environmental variable to the improvement of node purity in the SAMs (RF model), the importance of the variables used in the model was evaluated^49^. Three bioclimate variables (Bio1, Bio15, and Bio13) significantly contributed to the predictive ability of the SAMs. Four land type variables (Prop_grass, Prop_agri, Prop_forest, and Prop_urban) showed a relatively moderate contribution (Fig. 4A). Partial dependence plots presented complex responses of *V. v. nigrithorax* abundance relative to environmental gradients. Lower annual mean temperature, higher precipitation seasonality, and higher precipitation of the wettest month constrained the abundance of the species in Korea (Bio1, Bio15, and Bio13 in Fig. 4B). There were certain critical levels in determining the abundance in relation to land type proportions such as grassland, agricultural land, and urban area (Prop_grass, Prop_agri, and Prop_urban in Fig. 4B), whereas forest proportion showed a monotonical increment relationship (Prop_forest in Fig. 4B). The abundance of *V. v. nigrithorax* according to the proportion of barren land, water area, and wetland was relatively stable over the environmental gradient (Prop_barren, Prop_water, and Prop_wet in Fig. 4B). These variables showed lower contribution than others in the RF model (Fig. 4A).

**Figure 5:**
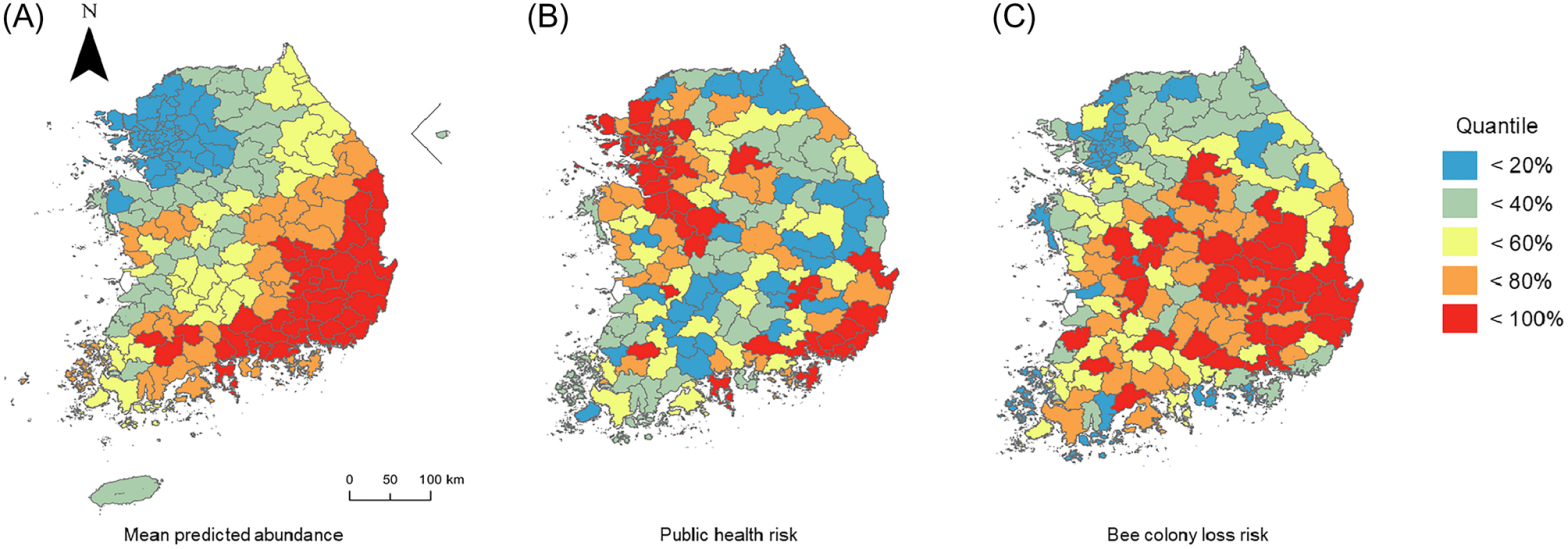
Predicted abundance classes of *Vespa velutina nigrithorax* (**A**), and human health risk (**B**), and honeybee loss risk (**C**) categorized into five levels. Residential population and number of honeybee colonies were considered as sensitivity factors of (**B**) and (**C**), respectively.

Both standardized NFA and questionnaire data showed positive linear correlations with our abundance estimates of *V. v. nigrithorax* aggregated as a given administrative unit (Fig. 5A) (NFA: Pearson’s r = 0.322; t = 4.268; df = 157; *p* < 0.0001, apiary: Pearson’s r = 0.629; t = 10.134; df = 157; *p* < 0.0001). Risk indices were projected into geographic space with sensitivity factors being the residential population and the number of honeybee colonies. Risk maps suggested that the main capital and southeast area would have more concerns about venomous stings for civilians and consequent hive removal efforts (Fig. 5B) (Supplementary Table 1). On the other hand, honeybee losses due to *V. v. nigrithorax* occurrence were predicted to be a concern centered in southern and central parts of Korea (Fig. 5C).

## Discussion

In this study, we developed a predictive model for estimating local abundance of *V. v. nigrithorax* in Korea, where the species has established across the entire study area after invasion. Our final model variant exhibited upper-moderate performance in comparison to the set of best models for 1547 species in a recent study that extensively evaluated performances of SAMs^34^. Subsequently, we estimated risk indices based on administrative areas to identify potential impacts of *V. v. nigrithorax*. Areas with high risk require measures to mitigate the impact of *V. v. nigrithorax*, such as density reduction and preemptive control measures before the risky season, taking into account the phenology and demographic processes of *V. v. nigrithorax*.

Among the environmental factors input into a final SAMs model, bioclimatic variables showed more significant contributions to model compared to land type variables. This partially indicates that lifecycle and reproduction processes of *V. v. nigrithorax* may be more influenced by climatic conditions than by the type of surrounding habitats^24^. Within the bioclimatic factor, Bio1 (annual mean temperature) was the most important variables, as suggested by SDMs model of Kim et al.^27^. The local abundance of *V. v. nigrithorax* generally had a positive correlation with annual mean temperature (Bio1), but abundance slightly decreased at the upper extreme of the annual temperature range in Korea (Table 2 and Fig. 4B). This trend partially consistent with an SDM study focused on the Iberian Peninsula, where *V. v. nigrithorax* was found in annual temperature range of 0.9–15.8 °C within the entire range of 0.9–18.5 °C in the study area. However, the Iberian study suggested a negative relationship between the hornet’s occurrence and the annual temperature variable. This discrepancy might arise if the changes in presence frequency, which did not provide specific abundance information, were not distinctly discernible within the given temperature range.

Precipitation-related variables in the summer season of Korea (Bio15, Bio13, and Bio12) were found to negatively influence the local abundance of *V. v. nigrithorax*. High precipitation in the summer might limit the foraging activities of worker hornets, and could potentially constrain feeding of *V. v. nigrithorax* larvae during their main population growth season^28,53^. Conversely, lower precipitation in the driest month, typically the winter season in Korea, was predicted to have a slight negative effect on the abundance of *V. v. nigrithorax*. Such dryness stress has also been suggested in previous SDMs studies^22,27,28^. This condition likely affects the successful overwintering of foundress queens, which start building a primary nest in the following spring season in Korea^8^. This aligns with a study that reported the positive influence of precipitation during the wettest month, typically winter in the Iberian Peninsula, on the establishment probability of *V. v. nigrithorax*^24^.

The partial dependence plots of Prop_grass and Prop_agri showed a distinctive inflection point (Fig. 4). After this point, the abundance of *V. v. nigrithorax* remained relatively low. A previous SDMs study found that the proportion of agricultural fields had a significant positive effect on the presence of *V. v. nigrithorax*, suggesting that this trend may result from the type of farming practices in the studied regions^24^. This research proposed that large areas with extensive farming in the study regions could provide a higher degree of heterogeneity, inclusive of natural habitats. In contrast, Korea generally exhibits intensive farming practices, such as rice paddy fields. This might imply that a high proportion of grassland and agricultural land with low heterogeneity in Korea could negatively impact *V. v. nigrithorax* abundance due to limited resources to support large colony populations. Furthermore, low heterogeneity in grassland and agricultural fields might impede new colony formation, considering that *V. v. nigrithorax* prefers to build their nests on forest stands. On the other hand, forest areas positively related the abundance. Thus, *V. v. nigrithorax* seems to be able to obtain sufficient resources for building nests and obtaining food from the forest landscape. Barren land, water bodies, and wetlands made only small contributions to the SAMs. These abundance responses according to the area of land types are roughly consistent with the result that the foraging radius of *V. v. nigrithorax* workers consisted of forest and semi-natural areas, agricultural areas, artificial areas, and water bodies in that order^7^.

Based on the investigation of *V. v. nigrithorax* across Korea for over 4 years, we were able to detect *V. v. nigrithorax* even in the main capital area. This observation supports the adaptability of *V. v. nigrithorax* to urban environment. However, we did not find evidence from its partial dependence plot to suggest a preference for highly urbanized areas. Rather, we found that its abundance was slightly decreased in locations where most foraging radii consisted of the urban area (Fig. 4B). Bessa et al.^26^ also reported *V. v. nigrithorax* could establish in area where urbanized proportion up to 79.7%, but not beyond. Similarly, Herrera et al.^28^ suggested that while anthropogenic factor (Human footprint variable) could increase the possibility of establishment, this effect declines at higher extreme. Therefore, the high incidence of nest removals and sting accidents by *V. v. nigrithorax* in urbanized areas could thus be due to a greater likelihood of encounters with citizens/residents and the general prevalence of the hornets. In fact, reports by citizens were more frequent during the season when the nests were clearly visible due to trees losing their leaves in autumn and hornets being most active (https://www.nfa.go.kr/).

With these findings, we still have the limitation on this SAM model. Firstly, there is a possibility that the spatial resolution (1 km^2^) of our input environmental variable set could not explain the demographic process of *V. v. nigrithorax,* which might be regulated at a finer scale. For example, the development rate of *V. v. nigrithorax* is probably affected by the milder condition in the nest because variation of the inside temperature of the nest is much less than the outside (unpublished, own observation). The climatic variable used in this study could implicitly incorporate energy dynamics of *V. v. nigrithorax* for maintaining the nest temperature, but may not directly reflect the effect of temperature on the development of the species. Secondly, our abundance data might be the result of a complex interaction between *V. v. nigrithorax* and competing species within limited resource availability. If strong competitors such as *V. mandarinia* are present in a certain region, new establishment and population growth of *V. v. nigrithorax* might be suppressed even though local environments are suitable for the species^54^. Therefore, covariate space should be extended using many other missing variables with finer scales related to *V. v. nigrithorax* for more accurate and specific abundance prediction.

Another consideration for more accurately predicting the abundance of *V. v. nigrithorax* involves the variation in demographic processes affected by annual environmental variation and invasion dynamics. In this study, we sampled the hornet during specific years rather than over a long term, but used average bioclimatic variables. This potentially introduced uncertainty in the abundance prediction, stemming from various components of the population process such as the survivorship of overwintering queens, successful nesting, food availability, and competing species during the active foraging season. Additionally, even though this hornet has already expanded its range throughout Korea, it is possible that the population has not yet reached an equilibrium state, a key precondition in ecological niche studies^30^. The equilibrium assumption has been violated in many studies on invasive species, due to the difficulties distinguishing between absence data and dispersal constraints^26,55^. As an alternative, so-called invasive species distribution models (iSDMs) can include other variables describing the time after invasion for the study area or constrain the spatial extent within an area where equilibrium can be assumed^26,56^. However, due to the lack of reliable data regarding the invasion time and the limited spatial extent of our study area, we did not consider the iSDM approach. Therefore, long-term monitoring is required to develop more stable SAMs that address variations in demographic processes as well as reliable SDMs. Despite these potential limitations, our model is more informative for assessing the impact of invaders as it provides direct abundance estimates, compared to the classical SDMs that generates the establishment probability of *V. v. nigrithorax* in Korea.

We empirically tested whether the classical SDMs could predict the abundance of *V. v. nigrithorax*. The occurrence model indicated that environmental conditions in Korea were mostly suitable for the persistence of *V. v. nigrithorax* as suggested by other studies^22,26,27^, showing 93% of the area having an establishment probability of more than 0.5. However, the performance in abundance prediction was significantly different between the two models adopting SAMs and SDMs. This difference might be caused by the form of the response variable^31^. The binary response used in the SDMs, constraints absence points regardless of abundance level and predicts establishment probability based on the frequency of presence points in the model (Fig. 2). If certain species are more abundant around the center of the niche (i.e., highest probability) estimated by the SDMs approach, it will be informative to predict abundance of the species^57,58^. However, our results showed no distinctive difference in the establishment probability of *V. v. nigrithorax* across the occupied area. This potentially indicates that a specific location with a higher suitability for establishment, within a given covariate space, may not always correspond to a higher density. Rather, our study supports the view that the relationship between occurrence-abundance is not evident or at least suggests that there is an oversimplification of the environmental niche estimated by binary occurrence data for *V. v. nigrithorax*^32,35,59^.

The mean abundance of *V. v. nigrithorax* in the administrative unit was able to explain the regional variation of hive removals and of the occurrence rate of this species in the apiary. Based on the abundance map, we suggested risk indices of human health and honeybee losses caused by *V. v. nigrithorax* in Korea, including sensitivity factors such as residential population and number of honeybee colonies in a given administrative area. Because of the sensitivity factor, human health risks were centered in the main capital area and southern part of Korea, which well matched with recorded hive removal calls in NFA data. Higher risks of honeybee losses were mainly present in the southeastern part (Gyeongsangnam-do) of Korea. These results were also consistent with previous reports, although the information for validation has a very coarse resolution at the province level. Because our risk function consisted of an estimated density of *V. v. nigrithorax* and sensitivities, it implies that risk could be managed by decreasing hazards through proper actions for controlling the local abundance of *V. v. nigrithorax*.

In summary, this study was conducted to develop a predictive model for the local abundance of *V. v. nigrithorax* in Korea. The abundance data of *V. v. nigrithorax* were obtained from field samplings from 253 sites for 4 years and used for model development with bioclimatic and land-type variables. Along with the SAMs, the classical occurrence model was also developed for comparison of local abundance prediction. With higher discriminative power and accuracy, the SAMs performed better in evaluating impacts caused by *V. v. nigrithorax*. Based on the final SAMs, risk indices for human health and honeybee losses were suggested. These results can provide support for risk management of *V. v. nigrithorax* in Korea, and offer biological information to other countries where this species has already become established or is likely to invade in the future. The methodology presented here can be adopted by other countries and targeted towards other pest species to provide valuable information.

### Supplementary Information

Supplementary Information.

## Data Availability

Data are available from the corresponding author upon reasonable request.
