# Supplementary material for: Modeling abundance and risk impact of Vespa velutina nigrithorax (Hymenoptera: Vespidae) in Korea: application of a species abundance model
Source: Sci Rep. 2023 Aug 21;13:13616. doi: 10.1038/s41598-023-40016-9 (PMC10442361; doi:10.1038/s41598-023-40016-9)
Supplement: Supplementary file 1 — Supplementary Information. [file 41598_2023_40016_MOESM1_ESM.docx]

**Modeling abundance and risk impact of *Vespa velutina nigrithorax* (Hymenoptera: Vespidae) in Korea: Application of a species abundance model**


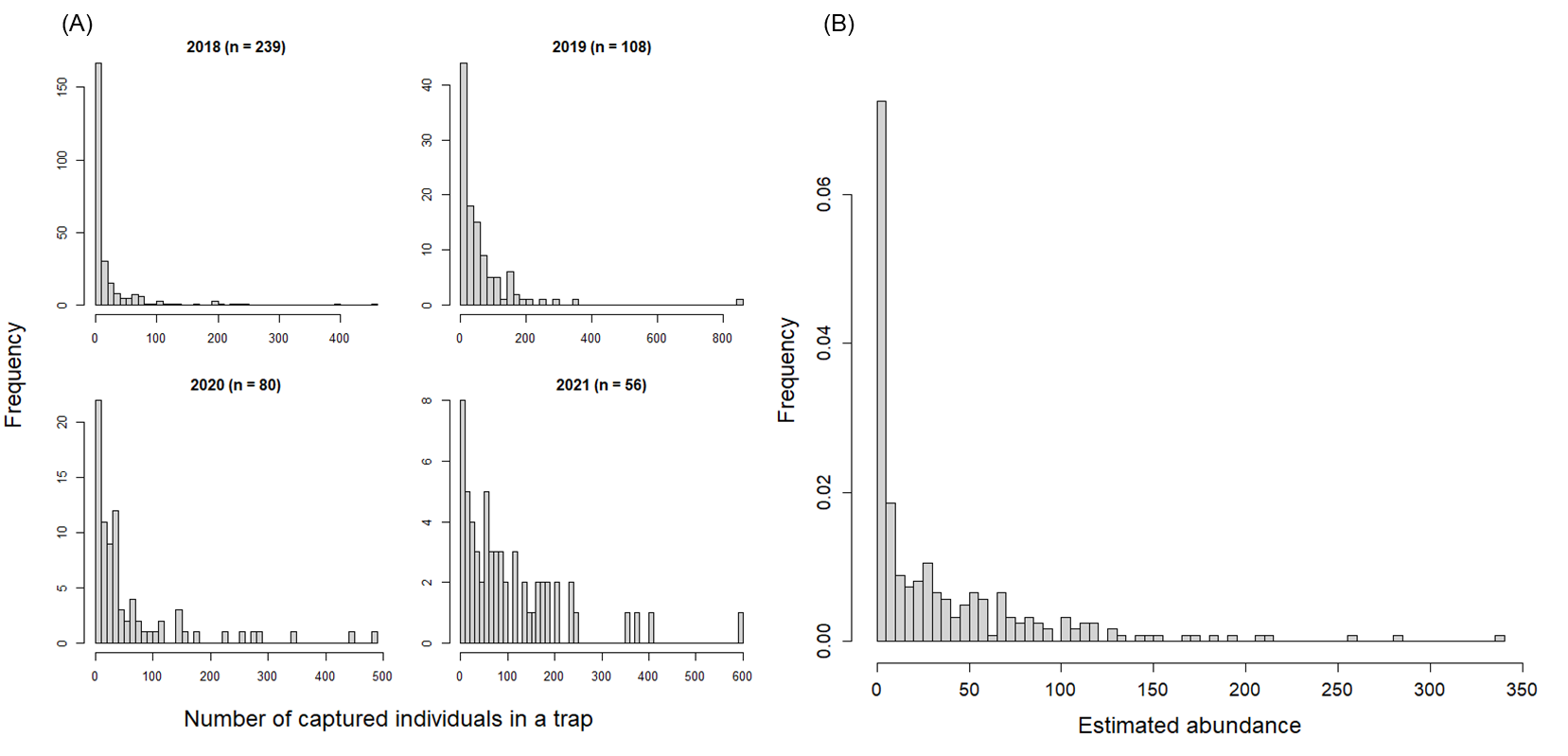


Supplementary Figure 1. (A) Distribution of *Vespa velutina nigrithorax* individuals captured each year and (B) Estimated abundance distribution, calculated as a four-year average of captures.

***
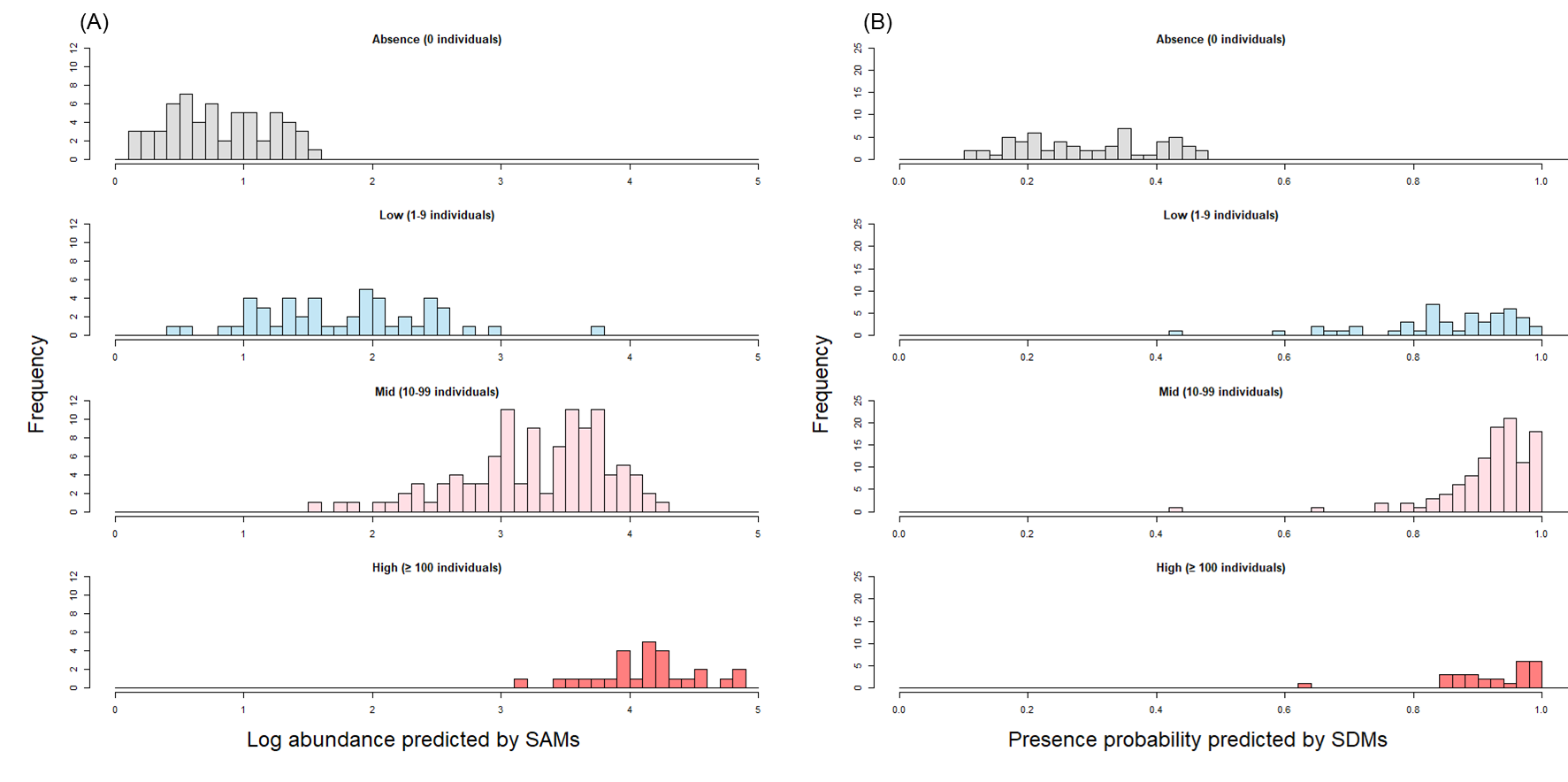
***

Supplementary figure 2. Distributions of predicted (A) log abundance and (B) establishment probability of *Vespa velutina nigrithorax* relative to observed abundance levels

Supplementary Table 1. Predicted mean abundance and associated factors used for calculating risk indices of *Vespa velutina nigrithorax*, summarized by administrative unit (City or County) in Korea.

| Province | City or County | Predicted mean abundance of *V. v. nigrithorax* | Sensitivity factor | |  | Standardized risk index (0-1) | |  | Observed impact | |
| --- | --- | --- | --- | --- | --- | --- | --- | --- | --- | --- |
|  |  |  | Residential population | No. honeybee colony |  | Health | Bee colony |  | Hive removal call  (NFA) | % appearance rate  (Apiary questionnaire) |
| GB | Daegu | 3.6 | 2,468,222 | 55,115 |  | 0.88 | 1.00 |  | 707 | 60 |
| GN | Ulsan | 3.5 | 1,168,469 | 34,587 |  | 0.40 | 0.61 |  | 318 | 94 |
| GB | Cheongdo-gun | 3.4 | 23,251 | 16,031 |  | 0.01 | 0.27 |  | 19 | 60 |
| GB | Gyeongju-si | 3.4 | 123,071 | 42,062 |  | 0.04 | 0.72 |  | 114 | 60 |
| GB | Chilgok-gun | 3.3 | 53,883 | 20,925 |  | 0.02 | 0.35 |  | 113 | 60 |
| GB | Gyeongsan-si | 3.3 | 124,869 | 25,986 |  | 0.04 | 0.43 |  | 30 | 60 |
| GB | Yeongcheon-si | 3.3 | 53,904 | 26,393 |  | 0.02 | 0.43 |  | 44 | 60 |
| GB | Pohang-si | 3.2 | 530,404 | 27,584 |  | 0.17 | 0.44 |  | 135 | 60 |
| GB | Yeongdeok-gun | 3.2 | 38,358 | 29,221 |  | 0.01 | 0.47 |  | 16 | 60 |
| GB | Gunwi-gun | 3.2 | 13,258 | 29,119 |  | 0.00 | 0.46 |  | 23 | 60 |
| GN | Miryang-si | 3.1 | 108,422 | 34,007 |  | 0.03 | 0.54 |  | 85 | 94 |
| GN | Changnyeong-gun | 3.1 | 65,120 | 29,314 |  | 0.02 | 0.46 |  | 46 | 94 |
| GN | Jinju-si | 3.1 | 352,553 | 34,770 |  | 0.11 | 0.54 |  | 220 | 94 |
| GN | Yangsan-si | 3.1 | 357,078 | 8,718 |  | 0.11 | 0.14 |  | 104 | 94 |
| GN | Haman-gun | 3.1 | 69,198 | 12,945 |  | 0.02 | 0.20 |  | 75 | 94 |
| JN | Hwasun-gun | 3.1 | 62,584 | 17,127 |  | 0.02 | 0.26 |  | 63 | 72 |
| GN | Uiryeong-gun | 3.1 | 27,933 | 14,583 |  | 0.01 | 0.22 |  | 54 | 94 |
| GN | Gimhae-si | 3.0 | 561,468 | 16,870 |  | 0.17 | 0.26 |  | 144 | 94 |
| GN | Changwon-si | 3.0 | 1,059,813 | 35,342 |  | 0.32 | 0.54 |  | 517 | 94 |
| GN | Sacheon-si | 3.0 | 115,342 | 12,790 |  | 0.03 | 0.19 |  | 84 | 94 |
| JN | Gokseong-gun | 3.0 | 27,508 | 9,075 |  | 0.01 | 0.14 |  | 40 | 72 |
| JN | Yeosu-si | 3.0 | 276,957 | 9,119 |  | 0.08 | 0.14 |  | 139 | 72 |
| JN | Gwangju | 3.0 | 1,469,884 | 47,677 |  | 0.44 | 0.72 |  | 653 | 72 |
| GN | Sancheong-gun | 3.0 | 36,025 | 45,498 |  | 0.01 | 0.69 |  | 94 | 94 |
| GB | Gumi-si | 3.0 | 182,645 | 36,170 |  | 0.05 | 0.54 |  | 189 | 60 |
| GB | Uljin-gun | 3.0 | 25,535 | 23,067 |  | 0.01 | 0.34 |  | 58 | 60 |
| GB | Yeongyang-gun | 2.9 | 9,055 | 12,805 |  | 0.00 | 0.19 |  | 29 | 60 |
| GN | Goseong-gun | 2.9 | 53,532 | 6,424 |  | 0.02 | 0.09 |  | 53 | 94 |
| GB | Cheongsong-gun | 2.9 | 25,623 | 11,556 |  | 0.01 | 0.17 |  | 15 | 60 |
| JN | Gwangyang-si | 2.9 | 151,796 | 18,303 |  | 0.04 | 0.27 |  | 82 | 72 |
| GN | Busan | 2.9 | 3,466,563 | 7,875 |  | 1.00 | 0.12 |  | 821 | 94 |
| GN | Hadong-gun | 2.9 | 47,099 | 13,511 |  | 0.01 | 0.20 |  | 40 | 94 |
| JN | Suncheon-si | 2.9 | 28,699 | 16,500 |  | 0.01 | 0.24 |  | 114 | 72 |
| GN | Tongyeong-si | 2.9 | 135,366 | 3,440 |  | 0.04 | 0.05 |  | 55 | 94 |
| GB | Seongju-gun | 2.9 | 23,621 | 62,908 |  | 0.01 | 0.91 |  | 56 | 60 |
| GB | Gimcheon-si | 2.9 | 68,393 | 28,325 |  | 0.02 | 0.41 |  | 126 | 60 |
| JN | Damyang-gun | 2.8 | 46,144 | 16,437 |  | 0.01 | 0.23 |  | 54 | 72 |
| JN | Boseong-gun | 2.8 | 39,292 | 25,539 |  | 0.01 | 0.36 |  | 68 | 72 |
| GB | Goryeong-gun | 2.8 | 16,565 | 17,237 |  | 0.00 | 0.25 |  | 83 | 60 |
| GB | Sangju-si | 2.8 | 101,678 | 50,258 |  | 0.03 | 0.71 |  | 80 | 60 |
| JN | Jangheung-gun | 2.8 | 36,514 | 3,594 |  | 0.01 | 0.05 |  | 26 | 72 |
| GB | Andong-si | 2.8 | 77,666 | 49,647 |  | 0.02 | 0.70 |  | 88 | 60 |
| GW | Samcheok-si | 2.8 | 68,001 | 13,156 |  | 0.02 | 0.18 |  | 77 | 31 |
| CC | Boryeong-si | 2.8 | 98,142 | 11,825 |  | 0.03 | 0.16 |  | 75 | 48 |
| JN | Gurye-gun | 2.8 | 25,173 | 11,019 |  | 0.01 | 0.15 |  | 24 | 72 |
| GN | Geoje-si | 2.7 | 256,578 | 5,746 |  | 0.07 | 0.08 |  | 69 | 94 |
| GB | Bonghwa-gun | 2.7 | 16,882 | 13,885 |  | 0.00 | 0.19 |  | 31 | 60 |
| CC | Cheongyang-gun | 2.7 | 30,396 | 22,902 |  | 0.01 | 0.31 |  | 26 | 48 |
| GB | Yecheon-gun | 2.7 | 56,165 | 20,841 |  | 0.01 | 0.28 |  | 70 | 60 |
| GN | Namhae-gun | 2.7 | 44,483 | 3,201 |  | 0.01 | 0.04 |  | 56 | 94 |
| JN | Goheung-gun | 2.7 | 62,631 | 17,277 |  | 0.02 | 0.23 |  | 52 | 72 |
| GW | Donghae-si | 2.7 | 91,453 | 9,035 |  | 0.02 | 0.12 |  | 79 | 31 |
| GN | Hapcheon-gun | 2.7 | 45,799 | 25,307 |  | 0.01 | 0.34 |  | 97 | 94 |
| JB | Sunchang-gun | 2.7 | 26,768 | 10,944 |  | 0.01 | 0.15 |  | 25 | 67 |
| GB | Uiseong-gun | 2.7 | 28,798 | 34,968 |  | 0.01 | 0.47 |  | 27 | 60 |
| GW | Taebaek-si | 2.6 | 44,124 | 2,798 |  | 0.01 | 0.04 |  | 42 | 31 |
| GB | Mungyeong-si | 2.6 | 37,069 | 18,394 |  | 0.01 | 0.24 |  | 43 | 60 |
| JN | Jangseong-gun | 2.6 | 43,343 | 10,777 |  | 0.01 | 0.14 |  | 68 | 72 |
| GB | Yeongju-si | 2.6 | 50,271 | 29,106 |  | 0.01 | 0.38 |  | 33 | 60 |
| CC | Gongju-si | 2.6 | 103,021 | 37,148 |  | 0.03 | 0.48 |  | 146 | 48 |
| JN | Gangjin-gun | 2.6 | 33,664 | 5,928 |  | 0.01 | 0.08 |  | 31 | 72 |
| CC | Seocheon-gun | 2.6 | 50,703 | 17,468 |  | 0.01 | 0.23 |  | 38 | 48 |
| JN | Sinan-gun | 2.6 | 38,096 | 2,513 |  | 0.01 | 0.03 |  | 2 | 72 |
| CC | Sejong-si | 2.6 | 346,275 | 16,446 |  | 0.09 | 0.21 |  | 85 | 48 |
| CC | Buyeo-gun | 2.6 | 63,599 | 27,371 |  | 0.02 | 0.35 |  | 58 | 48 |
| JB | Imsil-gun | 2.6 | 26,686 | 16,189 |  | 0.01 | 0.21 |  | 34 | 67 |
| GN | Hamyang-gun | 2.5 | 40,141 | 17,561 |  | 0.01 | 0.22 |  | 78 | 94 |
| JN | Jindo-gun | 2.5 | 29,996 | 10,384 |  | 0.01 | 0.13 |  | 11 | 72 |
| JB | Jangsu-gun | 2.5 | 21,683 | 7,682 |  | 0.01 | 0.10 |  | 22 | 67 |
| JB | Jeonju-si | 2.5 | 656,766 | 26,204 |  | 0.16 | 0.33 |  | 186 | 67 |
| GW | Gangneung-si | 2.5 | 215,911 | 12,469 |  | 0.05 | 0.16 |  | 131 | 31 |
| GW | Yeongwol-gun | 2.5 | 39,408 | 11,575 |  | 0.01 | 0.15 |  | 63 | 31 |
| JN | Wando-gun | 2.5 | 48,481 | 103 |  | 0.01 | 0.00 |  | 22 | 72 |
| JB | Jinan-gun | 2.5 | 24,957 | 17,151 |  | 0.01 | 0.22 |  | 17 | 67 |
| CC | Danyang-gun | 2.5 | 28,263 | 8,868 |  | 0.01 | 0.11 |  | 42 | 48 |
| GW | Yangyang-gun | 2.5 | 28,032 | 4,958 |  | 0.01 | 0.06 |  | 27 | 31 |
| JN | Hampyeong-gun | 2.5 | 31,157 | 16,769 |  | 0.01 | 0.21 |  | 25 | 72 |
| GW | Sokcho-si | 2.5 | 82,824 | 4,742 |  | 0.02 | 0.06 |  | 5 | 31 |
| JB | Namwon-si | 2.5 | 79,308 | 30,339 |  | 0.02 | 0.37 |  | 53 | 67 |
| JB | Wanju-gun | 2.5 | 91,060 | 37,302 |  | 0.02 | 0.46 |  | 85 | 67 |
| JN | Naju-si | 2.4 | 116,601 | 17,189 |  | 0.03 | 0.21 |  | 58 | 72 |
| CC | Daejeon | 2.4 | 1,493,979 | 32,144 |  | 0.35 | 0.39 |  | 436 | 48 |
| GW | Jeongseon-gun | 2.4 | 37,573 | 7,737 |  | 0.01 | 0.09 |  | 56 | 31 |
| GW | Goseong-gun | 2.4 | 28,392 | 6,415 |  | 0.01 | 0.08 |  | 24 | 31 |
| JN | Haenam-gun | 2.4 | 67,099 | 18,926 |  | 0.02 | 0.23 |  | 40 | 72 |
| JN | Muan-gun | 2.4 | 91,102 | 12,772 |  | 0.02 | 0.15 |  | 66 | 72 |
| JB | Muju-gun | 2.4 | 23,735 | 9,715 |  | 0.01 | 0.12 |  | 38 | 67 |
| JB | Gunsan-si | 2.4 | 265,160 | 4,909 |  | 0.06 | 0.06 |  | 66 | 67 |
| CC | Yeongdong-gun | 2.3 | 45,692 | 20,571 |  | 0.01 | 0.24 |  | 33 | 48 |
| GW | Pyeongchang-gun | 2.3 | 42,577 | 4,742 |  | 0.01 | 0.06 |  | 118 | 31 |
| CC | Goesan-gun | 2.3 | 37,525 | 22,590 |  | 0.01 | 0.26 |  | 34 | 48 |
| JN | Mokpo-si | 2.3 | 218,700 | 10,040 |  | 0.05 | 0.12 |  | 34 | 72 |
| CC | Gyeryong-si | 2.3 | 43,338 | 4,913 |  | 0.01 | 0.06 |  | 31 | 48 |
| GN | Geochang-gun | 2.3 | 62,726 | 25,658 |  | 0.01 | 0.30 |  | 58 | 94 |
| CC | Cheongju-si | 2.3 | 848,797 | 72,581 |  | 0.19 | 0.84 |  | 267 | 48 |
| GW | Inje-gun | 2.3 | 31,981 | 11,189 |  | 0.01 | 0.13 |  | 24 | 31 |
| CC | Boeun-gun | 2.2 | 31,871 | 20,511 |  | 0.01 | 0.23 |  | 75 | 48 |
| CC | Nonsan-si | 2.2 | 114,430 | 35,712 |  | 0.03 | 0.40 |  | 44 | 48 |
| CC | Hongseong-gun | 2.2 | 99,267 | 11,062 |  | 0.02 | 0.12 |  | 75 | 48 |
| CC | Jecheon-si | 2.2 | 131,407 | 20,147 |  | 0.03 | 0.22 |  | 69 | 48 |
| GW | Yanggu-gun | 2.2 | 23,052 | 8,893 |  | 0.01 | 0.10 |  | 32 | 31 |
| JB | Jeongeup-si | 2.2 | 106,534 | 28,973 |  | 0.02 | 0.32 |  | 58 | 67 |
| CC | Cheonan-si | 2.2 | 684,904 | 24,534 |  | 0.15 | 0.27 |  | 232 | 48 |
| JB | Iksan-si | 2.2 | 277,664 | 30,641 |  | 0.06 | 0.34 |  | 85 | 67 |
| JN | Yeonggwang-gun | 2.2 | 52,045 | 11,453 |  | 0.01 | 0.13 |  | 40 | 72 |
| JN | Yeongam-gun | 2.2 | 53,022 | 22,211 |  | 0.01 | 0.24 |  | 26 | 72 |
| CC | Okcheon-gun | 2.1 | 50,020 | 19,777 |  | 0.01 | 0.21 |  | 88 | 48 |
| CC | Yesan-gun | 2.1 | 76,703 | 15,791 |  | 0.02 | 0.17 |  | 56 | 48 |
| CC | Chungju-si | 2.1 | 209,368 | 39,722 |  | 0.04 | 0.43 |  | 84 | 48 |
| CC | Geumsan-gun | 2.1 | 50,335 | 15,567 |  | 0.01 | 0.16 |  | 67 | 48 |
| JB | Buan-gun | 2.1 | 50,772 | 12,124 |  | 0.01 | 0.13 |  | 78 | 67 |
| GW | Hwacheon-gun | 2.0 | 25,181 | 4,528 |  | 0.01 | 0.05 |  | 38 | 31 |
| GW | Hongcheon-gun | 2.0 | 70,065 | 9,048 |  | 0.01 | 0.09 |  | 109 | 31 |
| GW | Hoengseong-gun | 2.0 | 47,257 | 14,448 |  | 0.01 | 0.15 |  | 109 | 31 |
| JB | Gochang-gun | 2.0 | 53,366 | 39,684 |  | 0.01 | 0.40 |  | 73 | 67 |
| CC | Asan-si | 2.0 | 325,412 | 18,873 |  | 0.06 | 0.19 |  | 123 | 48 |
| GW | Wonju-si | 2.0 | 352,860 | 38,272 |  | 0.07 | 0.38 |  | 113 | 31 |
| CC | Taean-gun | 1.9 | 61,462 | 2,638 |  | 0.01 | 0.03 |  | 38 | 48 |
| JB | Gimje-si | 1.9 | 8,933 | 15,639 |  | 0.00 | 0.15 |  | 32 | 67 |
| GW | Cheorwon-gun | 1.9 | 46,295 | 9,691 |  | 0.01 | 0.09 |  | 24 | 31 |
| CC | Eumseong-gun | 1.9 | 92,113 | 23,286 |  | 0.02 | 0.22 |  | 57 | 48 |
| GG | Anseong-si | 1.8 | 194,954 | 14,344 |  | 0.03 | 0.13 |  | 63 | 39 |
| CC | Jincheon-gun | 1.8 | 85,458 | 13,870 |  | 0.02 | 0.13 |  | 58 | 48 |
| CC | Jeungpyeong-gun | 1.8 | 36,583 | 5,362 |  | 0.01 | 0.05 |  | 13 | 48 |
| GW | Chuncheon-si | 1.8 | 285,585 | 11,442 |  | 0.05 | 0.10 |  | 126 | 31 |
| CC | Dangjin-si | 1.8 | 167,050 | 19,265 |  | 0.03 | 0.17 |  | 127 | 48 |
| GG | Pyeongtaek-si | 1.8 | 537,135 | 12,690 |  | 0.09 | 0.11 |  | 120 | 39 |
| GG | Yeoncheon-gun | 1.7 | 46,407 | 5,413 |  | 0.01 | 0.05 |  | 38 | 39 |
| GG | Icheon-si | 1.7 | 223,349 | 7,826 |  | 0.04 | 0.07 |  | 86 | 39 |
| CC | Seosan-si | 1.7 | 176,630 | 13,116 |  | 0.03 | 0.11 |  | 74 | 48 |
| GG | Pocheon-si | 1.7 | 191,643 | 10,051 |  | 0.03 | 0.09 |  | 130 | 39 |
| GG | Gapyeong-gun | 1.7 | 63,936 | 11,556 |  | 0.01 | 0.10 |  | 99 | 39 |
| GG | Gwangju-si | 1.6 | 385,640 | 14,828 |  | 0.06 | 0.12 |  | 212 | 39 |
| GG | Yangpyeong-gun | 1.6 | 118,374 | 17,830 |  | 0.02 | 0.15 |  | 280 | 39 |
| GG | Yongin-si | 1.5 | 1,678,591 | 16,831 |  | 0.26 | 0.13 |  | 215 | 39 |
| GG | Paju-si | 1.5 | 465,612 | 17,812 |  | 0.07 | 0.14 |  | 76 | 39 |
| GG | Uiwang-si | 1.5 | 162,344 | 3,700 |  | 0.02 | 0.03 |  | 38 | 39 |
| GG | Incheon | 1.5 | 3,029,285 | 15,047 |  | 0.45 | 0.11 |  | 297 | 39 |
| GG | Hwaseong-si | 1.5 | 855,949 | 21,691 |  | 0.12 | 0.16 |  | 259 | 39 |
| GG | Yeoju-si | 1.4 | 114,659 | 11,707 |  | 0.02 | 0.08 |  | 81 | 39 |
| GG | Dongducheon-si | 1.4 | 98,245 | 1,514 |  | 0.01 | 0.01 |  | 58 | 39 |
| GG | Osan-si | 1.4 | 236,075 | 2,310 |  | 0.03 | 0.02 |  | 98 | 39 |
| GG | Suwon-si | 1.4 | 1,235,022 | 1,853 |  | 0.17 | 0.01 |  | 169 | 39 |
| GG | Seongnam-si | 1.3 | 960,342 | 8,956 |  | 0.13 | 0.06 |  | 74 | 39 |
| GG | Gunpo-si | 1.3 | 282,862 | 1,890 |  | 0.04 | 0.01 |  | 45 | 39 |
| GG | Anyang-si | 1.3 | 574,464 | 4,520 |  | 0.07 | 0.03 |  | 72 | 39 |
| GG | Namyangju-si | 1.3 | 709,307 | 9,426 |  | 0.09 | 0.06 |  | 226 | 39 |
| GG | Yangju-si | 1.3 | 229,778 | 5,326 |  | 0.03 | 0.03 |  | 125 | 39 |
| GG | Gwacheon-si | 1.2 | 58,462 | 1,387 |  | 0.01 | 0.01 |  | 15 | 39 |
| GG | Gimpo-si | 1.2 | 457,556 | 4,181 |  | 0.06 | 0.03 |  | 74 | 39 |
| GG | Ansan-si | 1.1 | 707,385 | 5,576 |  | 0.08 | 0.03 |  | 105 | 39 |
| GG | Uijeongbu-si | 1.1 | 447,026 | 4,010 |  | 0.05 | 0.02 |  | 50 | 39 |
| GG | Hanam-si | 1.1 | 274,734 | 6,006 |  | 0.03 | 0.03 |  | 37 | 39 |
| GG | Siheung-si | 1.0 | 508,379 | 3,788 |  | 0.05 | 0.02 |  | 99 | 39 |
| GG | Bucheon-si | 0.9 | 855,685 | 1,853 |  | 0.08 | 0.01 |  | 36 | 39 |
| GG | Goyang-si | 0.8 | 1,078,859 | 3,457 |  | 0.08 | 0.01 |  | 132 | 39 |
| GG | Gwangmyeong-si | 0.8 | 322,494 | 1,723 |  | 0.03 | 0.01 |  | 27 | 39 |
| GG | Seoul | 0.7 | 10,010,983 | 5,737 |  | 0.66 | 0.02 |  | 1339 | 39 |
| GG | Guri-si | 0.6 | 200,755 | 738 |  | 0.01 | 0.00 |  | 8 | 39 |
